# Pushing the Detailed Balance Limit in III–V Semiconductor Photoconversion with Bandgap-Engineering Multijunction Architectures

**DOI:** 10.3390/ma19020413

**Published:** 2026-01-21

**Authors:** Xing Gao, Yiming Yin, Boyu Yang, Chao Zhang, Wei Zhou, Jinchao Tong, Junhao Chu

**Affiliations:** 1 College of Smart Materials and Future Energy, Fudan University, Shanghai 200433, China; 24210300044@m.fudan.edu.cn (X.G.); yangby24@m.fudan.edu.cn (B.Y.); jhchu@fudan.edu.cn (J.C.); 2 Institute of Optoelectronics, College of Future Information Technology, Fudan University, Shanghai 200433, China; 25113090080@m.fudan.edu.cn (C.Z.); 25213080145@m.fudan.edu.cn (W.Z.); 3 Shanghai Xinyuan Innovation Center, Shanghai 200120, China; yinyiming@shhxinyuan.cn

**Keywords:** photoelectric conversion, photovoltaic, quantum well

## Abstract

The calculation of the limiting efficiency and structural optimization of solar cells based on the detailed balance principle is systematically investigated in this study. Through modeling and numerical simulations of various cell architectures, the theoretical efficiency limits of these structures under AM1.5G (Air Mass 1.5 Global) spectrum were quantitatively evaluated. Through a comprehensive consideration of the effects of bandgap and composition, the Al_0.03_Ga_0.97_As/Ge (1.46 eV/0.67 eV) cell configuration was determined to achieve a high theoretical efficiency of 43.0% for two-junction cells while maintaining satisfactory lattice matching. Furthermore, the study proposes that incorporating a Ga_0.96_In_0.04_As (8.3 nm)/GaAs_0.77_P_0.23_ (3.3 nm) strain-balanced multiple quantum wells (MQWs) structure enables precise bandgap engineering, modulating the effective bandgap to the optimal middle-cell value of 1.37 eV, as determined by graphical analysis for triple junctions. This approach effectively surpasses the efficiency constraints inherent in conventional bulk-material III–V semiconductor solar cells. The results demonstrate that an optimized triple-junction solar cell with MQWs can theoretically achieve a conversion efficiency of 51.5%. This study provides a reliable theoretical foundation and a feasible technical pathway for the design of high-efficiency solar cells, especially for the emerging MQW-integrated III–V semiconductor tandem cells.

## 1. Introduction

III–V multijunction solar cells have emerged as a pivotal research direction in high-efficiency photovoltaics. This is due to their tunable bandgaps, excellent carrier transport properties, and high internal quantum efficiencies. By integrating multiple subcells with optimized bandgaps, such architectures enable efficient conversion of distinct spectral regions and current matching. Such configuration significantly broadens the utilization of the solar spectrum [1]. This design substantially reduces carrier thermalization and transmission losses, ultimately leading to theoretical efficiency limits far exceeding those of single-junction cells. Shockley and Queisser’s seminal work in 1960 first applied the detailed balance principle to calculate the efficiency limit of single-junction solar cells [2]. Since then, this theoretical framework has served as the cornerstone for evaluating photovoltaic performance. Building upon this foundation, Henry systematically extended the theory to multijunction systems in the 1980s [3]. His work provided comprehensive modeling of their efficiency limits and catalyzed the critical transition of III–V multijunction solar cells from theoretical concept to technological reality.

Thanks to breakthroughs in advanced epitaxial technologies such as Metal–Organic Chemical Vapor Deposition (MOCVD) and Molecular Beam Epitaxy (MBE) [4,5], cell architectures have evolved from early three-junction designs to four-junction, five-junction, and even higher configurations [6]. Under laboratory conditions, III–V multijunction solar cells have reached the pinnacle of photovoltaic conversion efficiency. A certified efficiency of 39.5% has been achieved for a triple-junction (3J) cell under non-concentrated (1-sun) illumination [7], while a four-junction (4J) structure has attained a groundbreaking 47.6% under concentrated illumination (665×) [8]. These figures represent the highest recorded energy conversion levels achieved to date under both standard one-sun and concentrated photovoltaic (CPV) conditions. The remarkable performance potential of this material system and multijunction approach is highlighted.

In practical applications, III–V multijunction solar cells demonstrate distinctive advantages. Owing to their exceptionally high power-to-mass and power-to-area ratios, these cells are particularly suitable for area-constrained terrestrial applications [9]. Typical use cases include power systems for unmanned aerial vehicles, portable generators, and high-performance functional layers in building-integrated photovoltaics (BIPV). Notably, in the aerospace sector [10], III–V multijunction cells exhibit superior radiation resistance, high-temperature stability, and long-term operational reliability. These characteristics allow them to meet the stringent requirements of space missions for high efficiency, lightweight design, and extended service life. However, as the number of junctions increases, these cells still face numerous challenges in design and fabrication, including lattice constant matching, current balancing, and defect suppression. Therefore, systematically calculating efficiency limits and optimizing structures based on the detailed balance principle is essential. This approach not only enhances understanding of the underlying physics but also offers critical theoretical guidance and design principles. Such insights are vital for developing next-generation ultra-high-efficiency tandem solar cells.

This study systematically explores the efficiency limits and structural optimization of solar cells based on the detailed balance principle. We investigate and optimize single-junction, double-junction, and multijunction architectures. Through theoretical modeling and numerical simulation implemented in MATLAB R2023a, we evaluate the efficiency limits and loss mechanisms of various cell types under the AM1.5G spectrum. To further improve spectral utilization, this work also examines bandgap tuning via quantum well (QW) structures. This approach aims to tailor the absorption layer bandgap for targeted spectral matching, thereby overcoming the efficiency bottlenecks imposed by the bandgap limitations of traditional bulk materials. The findings are expected to provide theoretical foundations and practical design pathways for fabricating high-efficiency solar cells.

## 2. Model and Method

The idealized physical model for solar cells proposed by Shockley and Queisser is founded on the following key assumptions [2]: The bandgap energy (*E_g_*) of the material must satisfy *E_g_* > *kT* (where *T* is the ambient temperature) to ensure negligible thermal excitation of carriers at room temperature. The cell must be sufficiently thick to fully absorb all photons with energy greater than or equal to *E_g_*, while remaining completely transparent to photons with energy below *E_g_*. Each absorbed photon generates only one electron-hole pair, with excess energy from above-bandgap photons dissipated via thermalization, and no multiple exciton generation occurs. Carrier mobility is assumed infinite, with no transport losses, enabling complete collection of photogenerated carriers at the electrodes. The system follows the detailed balance principle, where radiative recombination is the only permitted mechanism, with no non-radiative recombination processes. Ideal ohmic contacts and zero surface recombination velocity are assumed, eliminating performance losses due to interfaces. These assumptions collectively form the theoretical foundation of the SQ efficiency limit for ideal solar cell efficiency.

**Figure 1:**
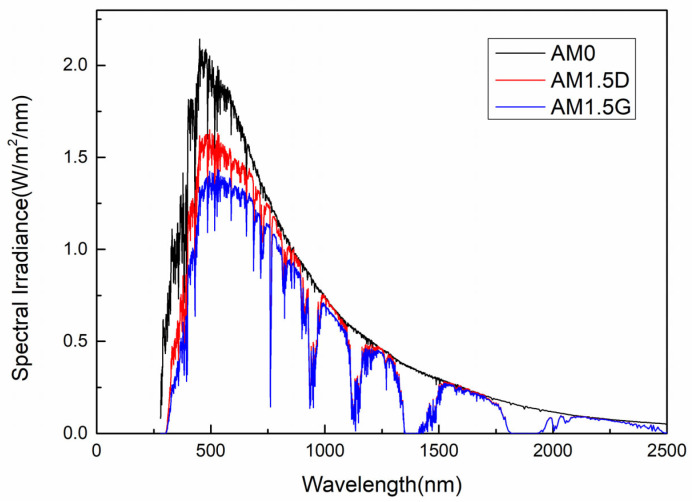
Comparison of the solar spectral irradiance distributions of AM0, AM1.5D, and AM1.5G.

The selection of the solar spectrum is decisive for the outcomes of theoretical modeling and efficiency calculations. Figure 1 plots and compares the spectral irradiance of the space solar cell standard spectrum AM0 (Air Mass 0) [11], AM1.5D (direct irradiance) [12], and the terrestrial standard spectrum AM1.5G (global irradiance) [13]. The AM0 spectrum is applicable to aerospace and satellite photovoltaic systems, representing solar radiation outside the Earth’s atmosphere. AM1.5D considers only the direct beam component and is used for evaluating the efficiency of concentrator photovoltaic systems. AM1.5G includes both direct and diffuse radiation, representing the total solar irradiance received at the Earth’s surface [14]. It serves as the international standard for assessing conventional terrestrial photovoltaic devices. The AM1.5G spectrum (with a total irradiance of 1000 W/m^2^) is attenuated in the UV region by ozone absorption, exhibits minor losses in the visible range due to Rayleigh scattering [15]. Additionally, characteristic absorption troughs appear in the infrared region due to water vapor and carbon dioxide. All subsequent theoretical calculations in this study are conducted under the AM1.5G spectral condition. As it more universally reflects the actual spectral response of terrestrial solar cells under non-concentrating environments [16], offering broader engineering reference value and comparative significance [17].

Under the assumptions of the Shockley–Queisser (SQ) theory, the photogenerated current density *J_ph_* for an n-junction tandem solar cell can be expressed as:

(1) $$J_{ph,1}=q\int_{0}^{\lambda_{1}}\frac{\lambda }{hc}I(\lambda )d\lambda \;(n=1),$$

(2) $$J_{ph,n}=q\int_{\lambda_{n-1}}^{\lambda_{n}}\frac{\lambda }{hc}I(\lambda )d\lambda \;(n>1),$$

where *q* is the elementary charge, λ  is the wavelength of light, *h* is Planck’s constant, *c* is the speed of light, and *I*(λ) denotes the spectral irradiance at wavelength λ under the AM1.5G solar spectrum. Let $u_{i}=E_{g,i}/kT\;(1\leq i\leq n)$, we can get

(3) $$J_{0,i}=\frac{2\pi qk^{3}T^{3}}{c^{2}h^{3}}e^{-u_{i}}(u_{i}^{2}+2u_{i}+2),$$

(4) $$V_{total}=\sum_{i=1}^{n}\frac{kT}{q}\ln\left(\frac{J_{ph,i}-J_{total}}{J_{0,i}}+1\right),$$

where *V_total_* is the overall voltage of the cell, numerically equal to the sum of the voltages of all subcells. *T* is the ambient temperature, taken as 300 K. k is the Boltzmann constant. *J*_0_ is the reverse saturation current density, and *J_total_* is the total current density of the cell.

In the SQ detailed balance-based model for tandem solar cells, the short-circuit current density *J_sc_* is a critical performance parameter. It is defined as the maximum current density deliverable by the cell under short-circuit conditions (*V* = 0), reflecting the number of photogenerated carriers that can be collected under illumination [18]. Its value is determined by the minimum photocurrent density among all subcells. This limitation arises because the subcells are connected in series. Under steady-state operation, the current flowing through the entire stack must remain uniform. As a result, the overall output current is constrained by the subcell yielding the smallest photocurrent. Any excess photogenerated carriers in the other subcells are dissipated through internal recombination. This fundamental constraint is known as the current matching condition, which acts similarly to a “bottleneck” effect, where the overall performance is limited by the weakest subunit. Under this constraint, the operating current density *J_total_* of the tandem solar cell ranges from 0 to *J_sc_*. When the external circuit is short-circuited, the output current density reaches this limiting value *J_sc_*. As the bias voltage increases, the current gradually decreases, achieving optimal power output at the maximum power point (*MPP*).

(5) $$J_{total}\leq J_{sc}=\min\{J_{ph,i}\},$$

This current matching principle not only dictates the ultimate efficiency limit of tandem solar cells, but also provides critical theoretical guidance for the selection of subcell bandgaps and the design of spectral splitting. In contrast to *J_sc_*, the open-circuit voltage *V_oc_* represents the maximum voltage attainable by the cell under open-circuit conditions (*J* = 0), and its theoretical upper limit is determined by the material bandgap. The fill factor (*FF*) is a key performance parameter that characterizes the “squareness” of the current–voltage output characteristic curve of a solar cell. It is defined as the ratio of the maximum power output (*P_mpp_*) to the product of *J_sc_* and *V_oc_*.

(6) $$FF=\frac{V_{mpp}\times J_{mpp}}{V_{oc}\times J_{sc}}$$

Here, *V_mpp_* and *J_mpp_* represent the voltage and current density at the maximum power point, respectively. FF physically reflects the capability of the solar cell to deliver maximum power. A higher FF value indicates that the current density-voltage (*JV*) curve more closely approximates a rectangle, implying lower losses during carrier transport and collection. Once *P_mpp_* is determined, the conversion efficiency (*Eff* or *η*) of the solar cell can be calculated using the following equation:

(7) $$Eff=\frac{P_{mpp}}{P_{in}}\times 100\%$$

The continuous exploration and enhancement of solar cell conversion efficiency hold significant theoretical value. It advances the frontiers of photonics and semiconductor materials science, and serves as a core driver for promoting photovoltaic technology iteration. Additionally, it contributes to reducing power generation costs and accelerating the transition of energy structures. As a key metric for evaluating the capability of photoelectric energy conversion, the efficiency limit is constrained by both the intrinsic properties of materials and fundamental thermodynamic principles. In this context, the SQ theory establishes a rigorous physical model and an efficiency ceiling for single-junction solar cells, forming the theoretical cornerstone of the field. This theory not only systematically elucidates the inherent mechanisms of spectral and radiative recombination losses but also provides an ideal reference framework for evaluating the performance of practical solar cells. Consequently, it guides critical research directions such as new material screening, bandgap engineering design, and the development of multijunction tandem technologies. Therefore, efficiency calculations based on SQ theory offer important theoretical guidance. Furthermore, they provide key physical insights and a clear optimization pathway, which are essential for empirical research and technological breakthroughs in high-efficiency solar cells.

To objectively assess the current state of photovoltaic technology, Table 1 summarizes the current efficiency records for various solar cell technologies under AM1.5G, as compiled and updated by leading research institutions. These data provide a critical benchmark for understanding the practical potential of different material systems and device architectures.

## 3. Results

### 3.1. Calculation of the SQ Efficiency Limit for Single-Junction Solar Cells

**Figure 2:**
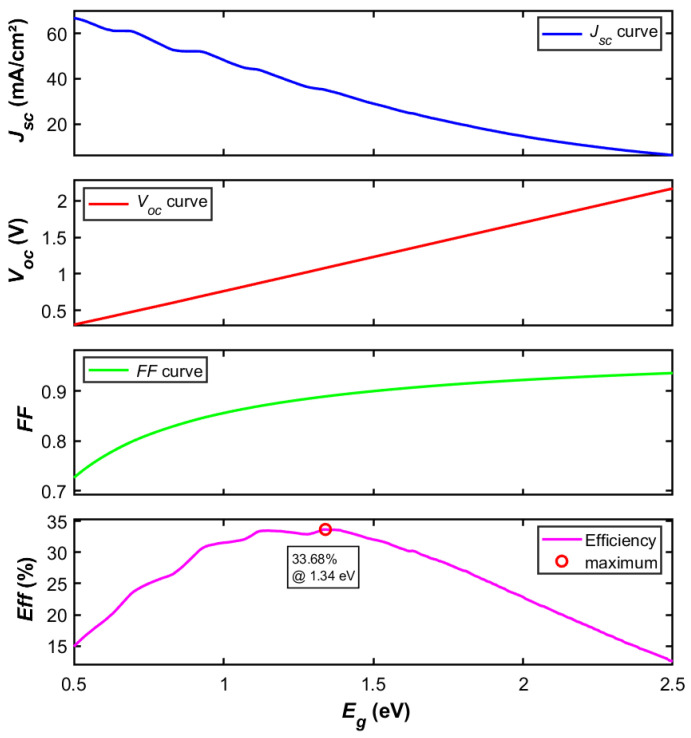
Dependence of *J_sc_*, *V_oc_*, *FF*, and *Eff* on bandgap energy in single-junction solar cells.

Within the Shockley–Queisser detailed balance framework and under its core assumptions, this study systematically calculated the bandgap-dependent performance parameters of single-junction solar cells. The evaluated metrics comprise short-circuit current (*J_sc_*), open-circuit voltage (*V_oc_*), fill factor (*FF*), and ultimate conversion efficiency (*Eff*). The computational results, illustrated in Figure 2, clearly reveal the theoretical performance limits and underlying physical mechanisms of single-junction devices. *J_sc_* exhibits a strong dependence on *E_g_*. As *E_g_* increases from 0.5 eV, *J_sc_* decreases monotonically. This trend stems directly from a fundamental assumption of the SQ theory, that is, only photons with energy *E* ≥ *E_g_* can be absorbed and contribute to the photocurrent. A larger *E_g_* narrows the range of the solar spectrum that can be effectively utilized, particularly resulting in the loss of low-energy photons in the long-wavelength region. Moreover, the number of high-energy photons in the AM1.5G spectrum is significantly lower than that of low-energy photons. Thus, an increase in *E_g_* leads to a continuous reduction in *J_sc_*_._ In contrast to the declining trend of *J_sc_*, Figure 2 shows that *V_oc_* increases approximately linearly with *E_g_*. This key behavior reflects the thermodynamic nature of the photovoltaic effect. *V_oc_* is determined by the splitting of quasi-Fermi levels under illumination, and its theoretical maximum approaches *E_g_*/*q*. Within the SQ model

(8) $$Voc\approx (\frac{kT}{q})\times \ln(\frac{Jsc}{J_{0}}+1)$$

In the SQ detailed balance theory, *J*_0_ represents the reverse saturation dark current density. It originates from photons with energies exceeding *E_g_* when the solar cell operates as a blackbody under thermal equilibrium conditions. Its value corresponds directly to the equivalent current density generated by radiative emission.

Although a decrease in *J_sc_* partially counteracts the increase in *V_oc_*, the exponential decay of *J*_0_ becomes overwhelmingly dominant when *T* = 300 K and *E_g_* > 0.5 eV. Calculations reveal that *V_oc_* increases in an approximately linear fashion with *E_g_*. It reflects the intrinsic advantage of wide-bandgap materials in suppressing thermodynamic spontaneous radiative losses. The *FF* is a monotonically increasing function of *E_g_*, primarily driven by the quasi-linear rise in *V_oc_*, though the rate of increase in *FF* gradually slows as *E_g_* becomes larger. The emergence of the maximum efficiency *Eff_max_* (~33.7% at *E_g_* = 1.34 eV) signifies the point where the decline in *J_sc_* begins to outweigh the gains from increasing *V_oc_* and *FF*. The bandgap of 1.34 eV represents the best compromise between absorbing a sufficient number of photons (high *J_sc_*) and minimizing the thermalization loss of high-energy photons (high *V_oc_*) in AM1.5G spectrum. This highlights the inherent limitation of single-junction structures in fully utilizing the solar spectrum and provides the fundamental theoretical rationale for the development of multijunction solar cells.

### 3.2. Calculation of the SQ Efficiency Limit for Two-Junction Solar Cells

The SQ theory clearly reveals that the efficiency of single-junction solar cells is fundamentally limited by the spectral mismatch of using a single bandgap material. Low-energy photons are lost due to transmission, while high-energy photons suffer from thermalization loss [34]. As a result, the theoretical peak efficiency is constrained to approximately 33.7%. To overcome this fundamental limitation, spectral splitting becomes essential. By stacking subcells with different bandgaps, high-energy photons are absorbed by the wide-bandgap top cell, and low-energy photons are collected by the narrow-bandgap bottom cell. III–V compound semiconductors, with their tunable bandgaps and excellent optoelectronic properties, serve as an ideal material system for realizing high-efficiency multijunction solar cells. In the series-connected architecture of multijunction solar cells, individual subcells are electrically connected via tunnel junctions. This implies that the total current density passing through the entire device is limited by the subcell generating the smallest photocurrent. Thus, current matching constitutes a central constraint for maximizing conversion efficiency. This section systematically investigates the SQ limiting efficiency of two-junction (2J) III–V solar cells based on the detailed balance principle. The focus is placed on tandem structures consisting of a tunable ternary compound top cell and a lattice-matched or nearly lattice-matched bottom cell. The evolution of key performance parameters with variations in the top cell composition *x* is analyzed, along with the underlying physical constraints.

**Figure 3:**
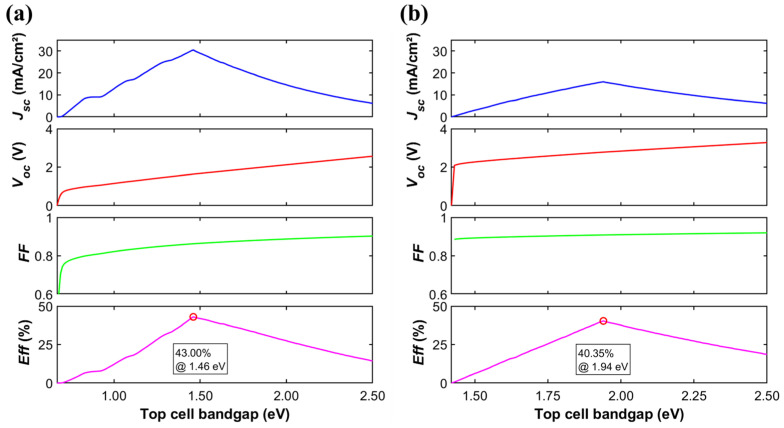
Dependence of *J_sc_*, *V_oc_*, *FF*, *Eff* on the top-cell bandgap in (**a**) Ge-based and (**b**) GaAs-based two-junction solar cells.

Figure 3 illustrates the systematic variation in key performance parameters with the top-cell bandgap for common Ge-based (*E_g_* = 0.67 eV) (Figure 3a) and GaAs-based (*E_g_* = 1.42 eV) (Figure 3b) two-junction solar cells. Both two-junction architectures exhibit pronounced bandgap-dependent characteristics. Both *J_sc_* and *Eff* display a single-peak trend as $E_{g}^{\mathrm{top}}$ increases, with the peak efficiency occurring at the same bandgap where *J_sc_* is maximized. This behavior stems from the current-matching constraint. When $E_{g}^{\mathrm{top}}$ is too low, the excess photocurrent generated in the top cell cannot be utilized by the bottom cell. When $E_{g}^{\mathrm{top}}$ is too high, reduced absorption of high-energy photons in the top cell leads to current loss. The peak position is determined by the critical condition of current balance between the subcells, i.e., $J_{\mathrm{sc}}^{\mathrm{top}}$ = $J_{\mathrm{sc}}^{\mathrm{bottom}}$. For instance, in a Ge-based tandem cell (Figure 3b), when $E_{g}^{\mathrm{top}}$ = 1.46 eV, the current-matched condition yields $J_{\mathrm{sc}}^{\mathrm{top}}$ = $J_{\mathrm{sc}}^{\mathrm{bottom}}$ = 30.45 mA/cm^2^, and the overall efficiency reaches approximately 43.0%. This result shows a notable efficiency improvement beyond the 33.7% theoretical limit of single-junction solar cells. The enhancement demonstrates that adding a series-connected subcell offers an effective approach to achieving higher solar energy conversion.

**Figure 4:**
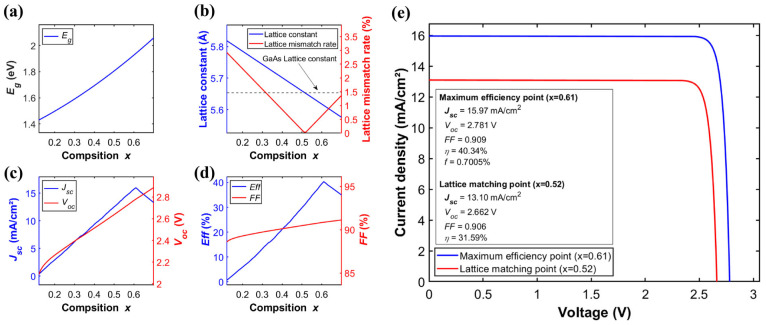
(**a**) *Eg* of Ga*_x_*In_1−*x*_P as a function of composition *x*. (**b**) Lattice constant of Ga*_x_*In_1−*x*_P and its lattice mismatch with GaAs versus composition *x*. (**c**,**d**) *J_sc_*, *V_oc_*, *Eff* and *FF* of the Ga*_x_*In_1−*x*_P/GaAs two-junction solar cell versus composition *x*. (**e**) *JV* characteristics of the Ga*_x_*In_1−*x*_P/GaAs cell at the lattice matching point and at the maximum efficiency point.

The lattice-matched GaInP/GaAs heterostructure is the dominant industrial configuration for III–V two-junction solar cells [19]. This technology has reached a high level of maturity, as demonstrated by its extensive and reliable long-term operation in spacecraft power systems, including satellites and deep-space probes. Figure 4. illustrates the correlation between the composition *x* in Ga*_x_*In_1−*x*_P (0.12 < *x* < 0.7) and its corresponding bandgap, lattice characteristics, and key optoelectronic parameters when employed as the top cell. The *Eg* of the Ga*_x_*In_1−*x*_P top cell increases monotonically with the gallium composition (*x*) (Figure 4a), while its lattice constant decreases linearly (Figure 4b). The electrical parameters exhibit a more complex dependence. The *J_sc_* (Figure 4c) and *Eff* (Figure 4d) both peak at *x* = 0.61, reaching 15.9 mA/cm^2^ and 51.5%, respectively. In contrast, *V_oc_* (Figure 4c) and *FF* (Figure 4d) show a monotonic increase with x.

(9) $$E_{g}^{GaInP}(x)=1.34+0.69x+0.48x^{2}(\mathrm{eV})$$

(10) $$a_{GaInP}=5.8687-0.4182x\;(Å)$$

In the design of GaInP/GaAs two-junction solar cells, the composition *x* directly governs both the bandgap and lattice constant of Ga*_x_*In_1−*x*_P. In such multijunction architectures, both materials maintain a zinc-blende (cubic ZnS-type) crystal structure, which offers the advantage of atomic-scale epitaxial compatibility. The consistent crystal symmetry between the top and bottom cells preserves tetrahedral bonding continuity and effectively suppresses the density of interfacial dangling bonds. When *x* < 0.7, Ga*_x_*In_1−*x*_P behaves as a direct bandgap semiconductor, with both the valence band maximum and conduction band minimum located at the Γ-point of the Brillouin zone [35]. This allows efficient photon absorption governed solely by energy conservation, eliminating the need for phonon-assisted momentum compensation. As a result, the material exhibits a high absorption coefficient α on the order of 10^5^ cm^−1^.

At *x* = 0.52, the top-cell GaInP is lattice-matched to the GaAs bottom cell with a lattice constant of 5.65 Å. However, its bandgap deviates from the optimal value. This results in a blue-shifted absorption edge and loss of longer-wavelength photons, limiting the conversion efficiency to 31.6%. In contrast, at *x* = 0.61, the bandgap is optimized, yielding an SQ limiting efficiency of 40.3%, albeit with a lattice mismatch (*f*) of 0.7%. In practical epitaxial processes, such a mismatch can introduce threading dislocations and defects. Designing multijunction cells thus involves a compromise between bandgap matching for spectral absorption optimization and lattice matching (to suppress interfacial recombination). Although *x* = 0.61 yields the highest efficiency in theoretical models, practical epitaxial growth requires strategies like strain-balanced superlattices or compositionally graded buffers. These techniques help mitigate lattice mismatch and enable performance closer to theoretical limits. Figure 4e. presents the *JV* characteristics and corresponding optoelectronic parameters of the GaInP/GaAs two-junction solar cell at both the lattice-matched point and the point of maximum efficiency.

**Figure 5:**
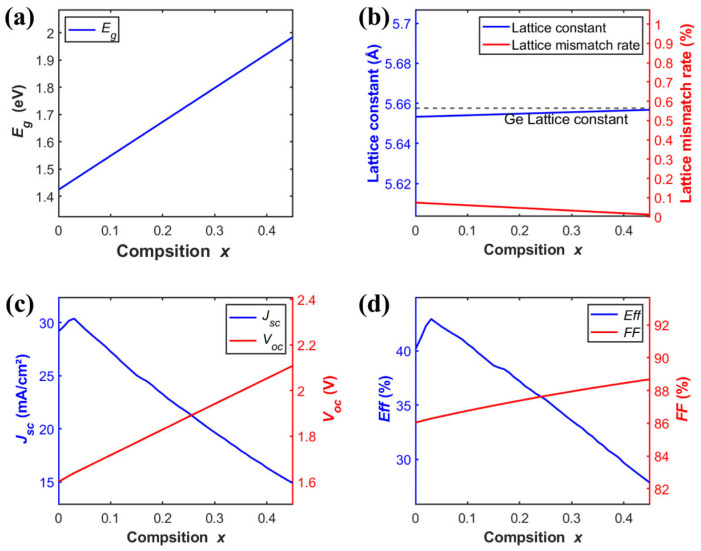
(**a**) *Eg* of Al*_x_*Ga_1−*x*_As as a function of composition *x*. (**b**) Lattice constant of Al*_x_*Ga_1−*x*_As and its lattice mismatch with Ge versus composition *x*. (**c**,**d**) *J_sc_*, *V_oc_*, *Eff* and *FF* of the Al*_x_*Ga_1−*x*_As/Ge two-junction solar cell versus composition *x*.

Ge-based solar cells offer a pivotal advantage as bottom subcells in multijunction architectures due to their overall performance. Their exceptionally low lattice mismatch with mainstream III–V materials such as GaAs and AlGaAs. It provides a foundation for high-quality epitaxial growth, enabling interfaces with low defect densities. The narrow bandgap of Ge extends the spectral response into the near-infrared (NIR) and short-wave infrared (SWIR) regimes up to ~1850 nm. Furthermore, Ge substrates exhibit excellent mechanical strength, high thermal conductivity, and benefit from well-established fabrication processes. These properties make Ge-based cells particularly suitable for space applications that demand high radiation tolerance and reliability in harsh environments. To this day, they continue to dominate the field of high-efficiency space photovoltaics. Adjusting the aluminum composition to 0.03 enables the tuning of $E_{g}^{\mathrm{top}}$ to 1.46 eV, which represents the theoretically optimal value for the top cell in a Ge-based two-junction solar cell (Figure 5a). This provides a physical foundation for achieving highly efficient current matching between the subcells. Furthermore, at this composition, the lattice constant of AlGaAs is approximately 5.6535 Å, resulting in a minimal lattice mismatch of only 0.07% with the Ge substrate (5.6575 Å) (Figure 5b). Such an extremely low mismatch facilitates the heteroepitaxial growth of high-quality top-cell crystalline material with low defect density. This effectively suppressing *V_oc_* losses caused by interfacial recombination (Figure 5c). As indicated in Figure 5d, this material system exhibits the potential to achieve a conversion efficiency of up to 43.0%.

**Figure 6:**
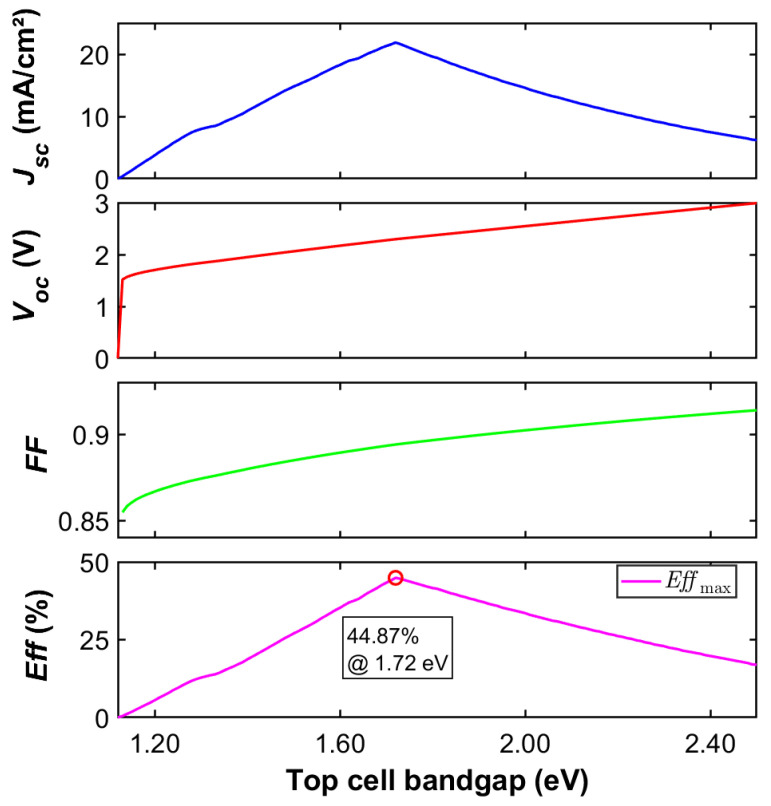
Dependence of *J_sc_*, *V_oc_*, *FF*, *Eff* on the top-cell bandgap in Si-based two-junction solar cells.

A tandem configuration using conventional silicon (Si) as the bottom-cell material can achieve a maximum theoretical efficiency of up to 44.9%. This occurs when the top-cell bandgap $E_{g}^{\mathrm{top}}$ is optimized to 1.72 eV, as illustrated in Figure 6. This peak efficiency originates from the nearly ideal spectral match between the two subcells, which significantly reduces thermalization losses across the solar spectrum. However, the practical integration of conventional Si into two-junction cells is severely limited by its intrinsic material properties. First, Si is an indirect bandgap semiconductor with a much lower absorption coefficient compared to direct bandgap materials. Consequently, a thickness of several hundred micrometers is necessary to fully absorb longer-wavelength photons. This requirement contradicts the multijunction design principle of using thin, highly efficient active layers. This substantial thickness also promotes bulk recombination losses. Second, Si exhibits significant lattice mismatch and thermal expansion coefficient mismatch with high-efficiency III–V top-cell materials. These mismatches induce high densities of threading dislocations at the heterointerface during epitaxial growth, forming severe non-radiative recombination centers that drastically degrade the *V_oc_*.

Therefore, in practical device architectures, Si is typically not utilized as a direct electricity-generating active layer, but rather serves as a mechanical support substrate and crystalline template for epitaxy [36]. To achieve this, a series of advanced material engineering techniques must be employed. Strain-compensated superlattice buffers (e.g., GaP/GaInP) gradually transition the lattice constant to suppress misfit dislocations. Low-temperature growth combined with thermal cycle annealing mitigates thermal stress-induced warping. Atomic-level surface planarization and passivation (e.g., via SiO_2_/Al_2_O_3_ stacks) suppress interfacial recombination [37]. Collectively, these processes enable Si to serve primarily as a robust substrate support in III–V/Si multijunction cells.

In laboratory designs, optimal bandgap alignment is prioritized to pursue higher conversion efficiencies. In such cases, techniques like buffer layers are implemented to mitigate the resulting lattice mismatch. In commercial manufacturing, economic considerations often take precedence; it is common to accept a modest efficiency compromise by prioritizing lattice-matched growth to streamline the manufacturing process and enhance device yield and reliability.

### 3.3. Calculation of the SQ Efficiency Limit for Triple-Junction Solar Cells

Building upon the systematic investigation of the performance limits and material constraints of two-junction solar cells, the research scope naturally extends to higher-order multijunction architectures. Incorporating a third subcell enables finer spectral splitting and utilization of sunlight, with a theoretical efficiency limit significantly surpassing that of two-junction configurations. The design principles of III–V triple-junction solar cells are discussed in this section, with a focus on optimizing bandgap combinations, implementing current matching strategies, and applying lattice matching techniques. The analysis aims to clarify their potential for nearing theoretical efficiencies and the challenges that must be addressed. A systematic analysis was conducted on the theoretical performance of triple-junction solar cells with GaAs as the fixed middle subcell. By systematically varying $E_{g}^{\mathrm{top}}$ and $E_{g}^{\mathrm{bottom}}$, the ultimate conversion efficiency of this triple-junction architecture was calculated. A two-dimensional contour map of efficiency as a function of $E_{g}^{\mathrm{top}}$ and $E_{g}^{\mathrm{bottom}}$ was subsequently generated. This contour plot clearly reveals the optimal bandgap combination corresponding to the peak efficiency. Furthermore, the *JV* characteristic curve corresponding to this optimal bandgap combination is provided to visually illustrate key parameters such as *MPP*.

**Figure 7:**
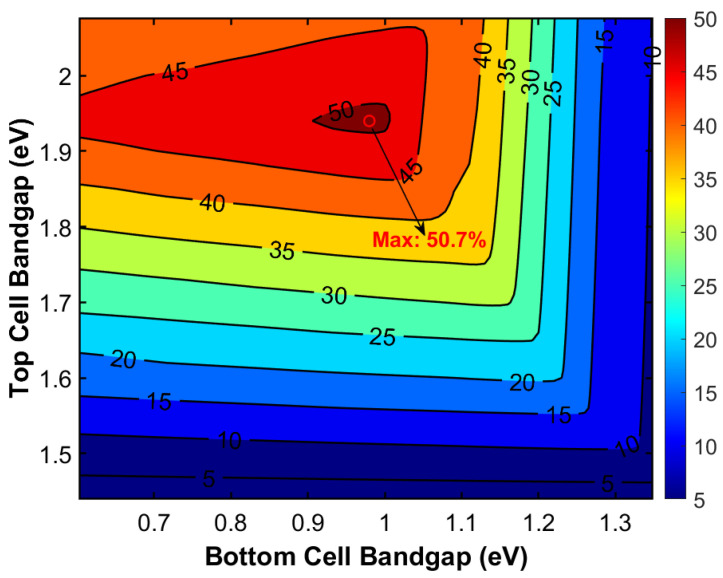
Efficiency landscape of a triple-junction solar cell with a fixed GaAs middle cell.

Due to its direct bandgap characteristic, GaAs demonstrates an extremely high absorption coefficient and outstanding carrier transport properties [38], which contribute to a high theoretical radiative recombination efficiency limit. Its bandgap energy aligns with the peak region of the solar spectral irradiance, enabling efficient absorption and conversion of photons at wavelengths around ~873 nm. Theoretically, this makes it an ideal bridge connecting wide-bandgap top cells and narrow-bandgap bottom cells. In a triple-junction architecture with GaAs as the middle cell, the structural design demonstrates significant integration advantages compared to more complex multijunction schemes. This configuration typically employs III–V materials with similar lattice constants—such as a GaInP top cell and a GaInAs bottom cell—and is monolithically grown in a single epitaxial process [39]. This avoids the need for complex bonding techniques or buffer layer designs required in mechanical stacking. Such on-chip integration greatly simplifies device fabrication, reduces optical and electrical losses at interconnected interfaces, and enhances intrinsic device reliability. Based on the computational results presented in Figure 7, the optimal bandgap combination with GaAs as the middle cell is determined to be 1.94 eV, 1.42 eV, and 0.98 eV, corresponding to an *Eff* of 50.7%. Precise adjustment of the composition x in ternary compounds such as Ga*_x_*In_1−*x*_P and Ga*_x_*In_1−*x*_As allows continuous tuning of their bandgap energies, enabling the co-design of the top and bottom cell bandgaps. Theoretical calculations indicate that a Ga_0.61_In_0.39_P/GaAs/Ga_0.67_In_0.33_As combination achieves optimal bandgap matching in a GaAs-middle-cell triple-junction solar cell.

**Figure 8:**
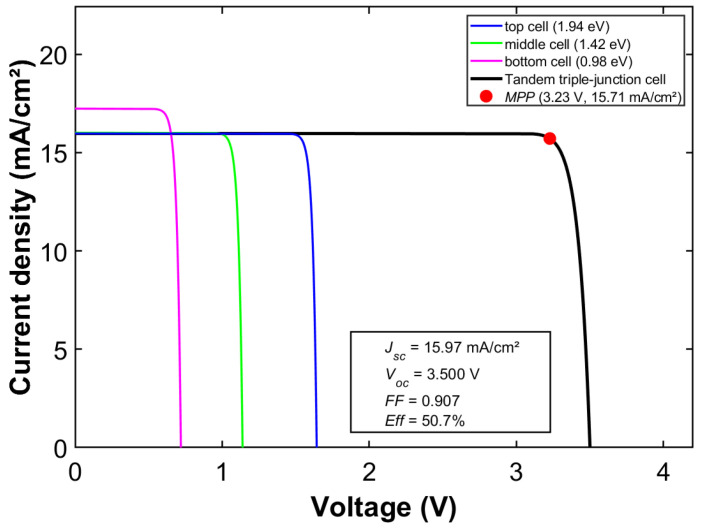
Detailed electrical performance under the optimal bandgap combination with a GaAs middle cell.

The detailed electrical performance under this optimal combination is shown in Figure 8. The J-V characteristic curve demonstrates that this bandgap configuration efficiently covers the spectral range from 300 to 1240 nm and achieves good current matching among the subcells. It allows the theoretical conversion efficiency of the triple-junction cell to approach the SQ limit.

The top cell Ga_0.61_In_0.39_P and the middle cell GaAs achieve excellent lattice matching (*f* < 1%), ensuring high crystal quality and low interfacial recombination rates in this region. However, a significant lattice mismatch (*f* > 2%) exists between the GaAs middle cell and the Ga_0.67_In_0.33_As bottom cell. Direct epitaxial growth would introduce a high density of threading dislocations, severely degrading device performance. To overcome this inherent lattice constraint, the inverted metamorphic multijunction (IMM) technology is typically employed [40]. A compositionally graded buffer (CGB) layer is grown between the GaAs middle cell and the underlying GaInAs bottom cell [10]. This buffer enables a continuous transition of the lattice constant, effectively confining the misfit strain within itself and promoting the lateral relaxation of mismatch dislocations. This process prevents the propagation of defects into the active regions, allowing the subsequent epitaxial growth of a high-quality GaInAs bottom cell with low dislocation density. The IMM structure breaks the traditional limitation of lattice-matching growth on GaAs substrates, enabling the free selection of material systems with more optimal bandgap combinations. This enables broader spectral coverage and higher-efficiency absorption and conversion of sunlight. Theoretically, this approach enables the conversion efficiency of the triple-junction solar cell to approach its detailed balance limit. Thus, it demonstrates the critical role of bandgap engineering and defect engineering in developing high-efficiency multijunction cells.

**Figure 9:**
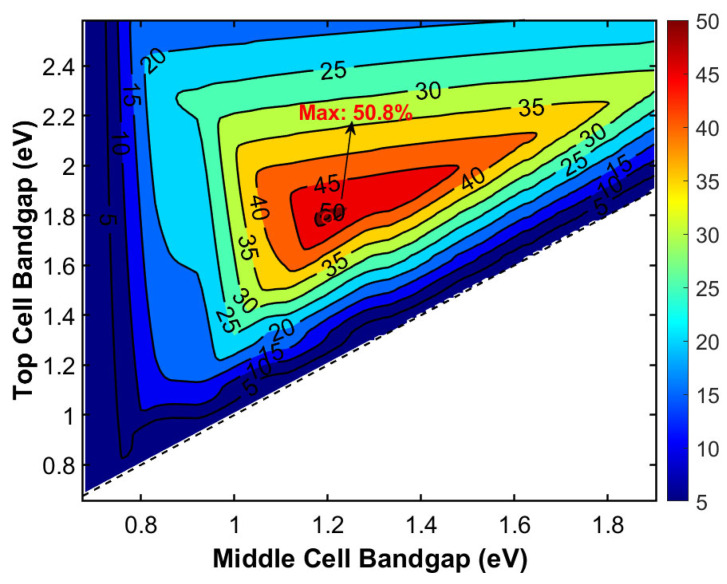
Efficiency landscape of a triple-junction solar cell with a fixed Ge middle cell.

We also conducted theoretical calculations and performance analysis of a triple-junction solar cell with Ge as the fixed bottom subcell. By continuously adjusting $E_{g}^{\mathrm{top}}$ and $E_{g}^{\mathrm{mid}}$, the theoretical limit efficiency of this triple-junction cell under AM1.5G spectrum was calculated. Figure 9 presents the resulting two-dimensional efficiency contour plot as a function of $E_{g}^{\mathrm{top}}$ and $E_{g}^{\mathrm{mid}}$. The contour clearly reveals the optimal bandgap combination region for achieving maximum efficiency.

**Figure 10:**
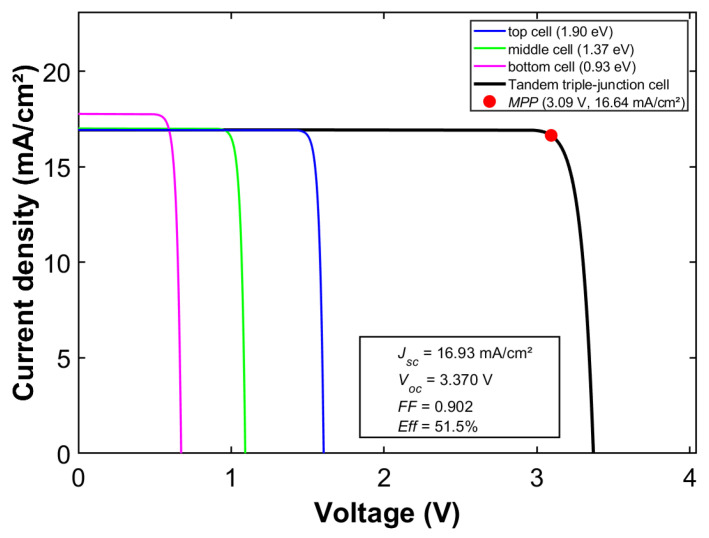
Detailed electrical performance under the optimal bandgap combination with a Ge middle cell.

Furthermore, Figure 10 presents the corresponding *JV* characteristic curve for this optimal bandgap combination, explicitly demonstrating the output characteristics of the triple-junction cell at the *MPP*. This provides an intuitive basis for understanding the electrical behavior of multijunction solar cells. It clearly demonstrates the key advantage of the germanium-based bottom subcell in enabling ultra-high conversion efficiency. When employing Ga_0.49_In_0.51_P (*E_g_* = 1.79 eV, a = 5.668 Å) as the top cell and Ga_0.85_In_0.15_As (*E_g_* = 1.20 eV, a = 5.714 Å) as the middle cell, this cell structure achieves a theoretical limit efficiency of 50.8% under AM1.5G spectrum. This performance is comparable to that of the optimal bandgap configuration using GaAs as the middle subcell, demonstrating excellent current matching and spectral response characteristics.

(11) $$E_{g}^{GaInAs}(x)=0.36+0.63x+0.43x^{2}(\mathrm{eV})$$

(12) $$a_{GaInAs}=6.0583-0.405x\;(Å)$$

The Ga_0.49_In_0.51_P/Ga_0.85_In_0.15_As/Ge triple-junction solar cell demonstrates a theoretical efficiency potential of 50.8%. However, the lattice mismatch between the top and middle cells (*f* ≈ 0.8%) and between the middle cell and the Ge substrate (*f* ≈ 1.07%) introduces high-density threading dislocations during epitaxial growth. These defects significantly degrade the open-circuit voltage (V_OC_) and fill factor (FF), presenting major technical obstacles to realizing this level of efficiency. To realize the target theoretical efficiency of the above triple-junction solar cell, a series of sophisticated epitaxial techniques must be employed to overcome the substantial lattice mismatch. These techniques primarily include growing compositionally graded buffer layers (e.g., GaInAs or GaAsP systems) on the Ge substrate to achieve continuous lattice constant transition and strain relaxation. Low-temperature buffer layers and superlattice dislocation filtering techniques are utilized to suppress the propagation of threading dislocations into active regions [41]. Thermal cycle annealing processes are incorporated to promote dislocation annihilation and recombination, ultimately yielding high-quality middle and top cell materials. Through these specialized processes, the exceptional mechanical, thermal, and optoelectronic properties of the Ge substrate can be leveraged to create a platform for ultrahigh-efficiency devices. It is an ideal choice for high-performance multijunction solar cells.

**Figure 11:**
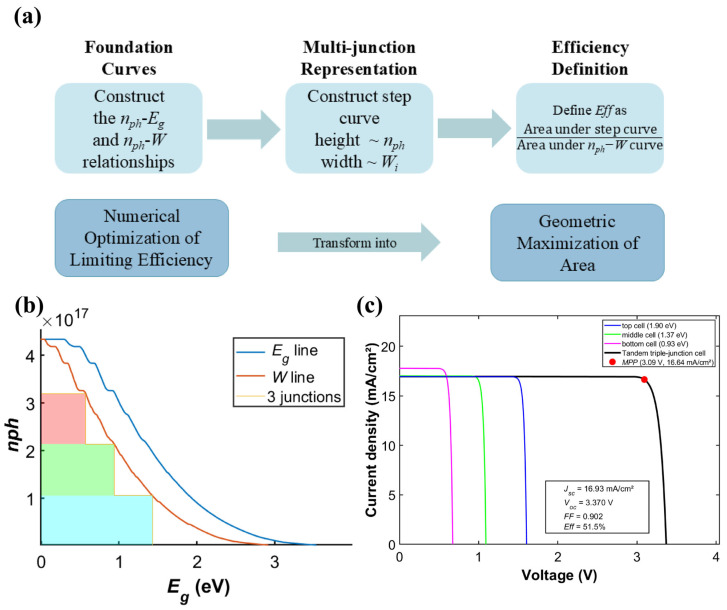
Graphical analysis of an optimal triple-junction solar cell: (**a**) conceptual schematic, (**b**) spectral absorption, and (**c**) electrical characteristics.

Following systematic investigation of the efficiency characteristics of two-junction systems with fixed subcells, the research focus has shifted toward seeking the global optimum for triple-junction solar cells. The conclusions of fixing a single junction are constrained by predefined material parameters and fail to capture the full optimization space of bandgap combinations for the whole structure. Therefore, graphical analysis is utilized to jointly optimize the bandgaps of all three subcells with an integrated viewpoint [3]. It facilitates progression toward the detailed balance limit efficiency of triple-junction solar cells. This graphical analysis has been used for analyzing multijunction solar cells. In this method, the photon flux (*n_ph_*) is plotted on the vertical axis and the maximum available work per photon (*W*) on the horizontal axis. For a triple-junction solar cell, the maximum efficiency corresponds to the largest area enclosed by a three-step staircase shape. The height of each step corresponds to the photon flux absorbed by the respective subcell, while its width represents the useful work output (*W*) per photon delivered by that subcell. The starting position of each step is determined by the bandgap energy and radiative recombination losses of the subcell. In practical optimization, current matching, which is achieved when each subcell absorbs an equal photon flux, is typically adopted as the central condition. Using this constraint, the optimal bandgap combination for the triple-junction cell can be determined. The corresponding maximum enclosed area of the staircase plot identifies the bandgap set that yields the highest theoretical efficiency for the entire device. The corresponding schematic diagram is presented in Figure 11a.

This method transforms complex multi-dimensional parameter optimization into an intuitive geometric area maximization problem. It also clearly reveals that multijunction cell efficiency is determined by solar spectrum shape, carrier thermalization loss, and radiative recombination limit. Therefore, transitioning from local optimization with fixed subcells to global optimization via graphical analysis is crucial. This transition is essential for thoroughly evaluating and achieving the maximum performance potential of triple-junction solar cells.

The optimal bandgap combination for a triple-junction solar cell was obtained using the graphical analysis method [3]. Corresponding performance characterization diagrams were subsequently plotted in Figure 11b. The total area of the three rectangular steps represents the maximum theoretical output work. The bandgap combination that maximizes this total area can be determined by adjusting the bandgap of the three subcells. This adjustment must ensure that each subcell absorbs an equal photon flux, thereby achieving current matching. The enclosed area reaches its maximum when the bandgaps are set to 1.90 eV, 1.37 eV, and 0.93 eV, respectively. This result indicates that this specific combination represents the global optimal solution for the triple-junction cell. The corresponding theoretical efficiency limit for this configuration is 51.5%. Figure 11c further presents the *JV* characteristic curve of the triple-junction solar cell under this optimal bandgap combination. The graph clearly demonstrates excellent current matching characteristics among the three subcells at the *MPP*, indicating effective distribution of photon absorption across the different bandgaps. The total voltage of the entire cell equals the sum of the voltages of three subcells., while *FF* remains at a high level. These results not only validate the effectiveness of the graphical analysis method at the theoretical level, but also confirm that the bandgap combination obtained through global optimization exhibits superior electrical performance. This outcome provides clear theoretical guidance and optimization directions for experimental design and material selection.

In the globally optimal bandgap combination obtained through graphical analysis, the target bandgaps of the top and bottom subcells can be achieved by adjusting the composition of conventional III–V ternary compounds (e.g., Ga_1−x_In_x_P, GaIn_1−x_As_x_). However, the target bandgap of 1.37 eV for the middle subcell falls within the unachievable range of traditional GaAs-based material systems. Although incorporating elements such as N (e.g., GaAsN) can reduce the bandgap, it inevitably introduces lattice mismatch [42]. This leads to dislocation proliferation and increased non-radiative recombination, severely limiting cell performance. To address this, we embed strain-balanced multiple quantum wells (MQWs) in the intrinsic region of the middle subcell [43]. This structure alternates compressively strained GaInAs quantum wells with tensile-strained GaAsP barrier layers [19]. The coupling of quantum confinement and strain effects enables effective tuning of the equivalent absorption bandgap, while maintaining overall lattice matching with the GaAs substrate. By designing the thickness, composition, and periodicity of the quantum wells and barriers, the effective bandgap of the middle subcell can be precisely adjusted to the target value of approximately 1.37 eV. This enables current matching and efficiency maximization for the entire triple-junction solar cell without compromising material quality.

**Figure 12:**
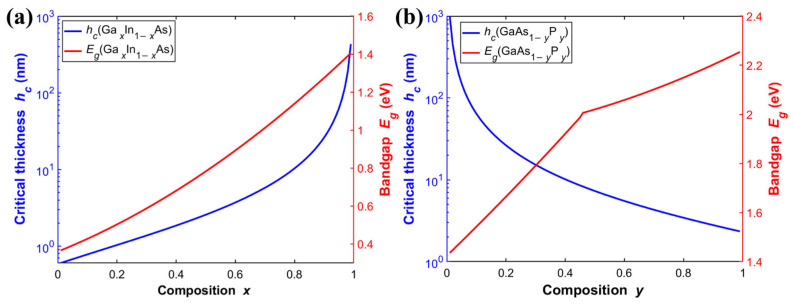
Variation in critical thickness and bandgap with composition for GaAs-based (**a**) Ga*_x_*In_1−*x*_As (**b**) GaAs_1−*y*_P*_y_*.

The critical thickness (*h_c_*) defines the maximum allowable thickness of a pseudomorphic strain layer before it relaxes and generates misfit dislocations during epitaxial growth [44]. If the thickness of a single strained layer exceeds its *h_c_*, the accumulated elastic strain energy will be released through the formation of crystal defects such as misfit dislocations, leading to severe non-radiative recombination. This causes significant degradation of the solar cell’s *V_oc_* and *FF*. Therefore, any strain-based band engineering must strictly control the thickness of each layer within *h_c_*, as determined by the material composition and strain magnitude. Based on the Matthews mechanical equilibrium model, the following equation quantitatively describes the *h_c_* for a specific GaInAs/GaAsP quantum well system, providing a reliable theoretical constraint for the design of MQWs structures [45]. Figure 12 clearly shows *h_c_* for GaAs-based Ga*_x_*In_1−*x*_As and GaAs_1−*y*_P*_y_*. The *h_c_* of Ga*_x_*In_1−*x*_As increases with the Ga composition (*x*) (Figure 12a), whereas the *h_c_* of GaAs_1−*y*_P*_y_* decreases as the P composition (*y*) increases (Figure 12b).

(13) $$h_{c}=\frac{a(1-\frac{C_{12}}{4(C_{11}+C_{12})})}{2\sqrt{2}\pi \frac{a_{0}-a}{a}(1+\frac{C_{12}}{C_{11}+C_{12}})}\ln(\frac{\sqrt{2}h_{c}}{a}+1)$$

where C_11_ and C_12_ are the elastic stiffness constants, and a_0_ and a represent the relaxed lattice constants of the substrate and the well/barrier layer, respectively.

After clarifying the *h_c_* of each strained layer, introducing a strain-balancing design criterion becomes essential. This approach is necessary to enable the controllable fabrication of multi-period quantum well structures and to prevent performance degradation resulting from strain relaxation. Even if the thickness of each layer remains below its critical thickness, the accumulated strain energy from multilayer stacking can still destabilize the entire structure. It leads to the formation of threading dislocations. Therefore, merely satisfying the single-layer critical thickness condition is insufficient to ensure the long-term stability and high efficiency of the device. Strain balancing addresses this issue by incorporating strain layers with opposite signs of stress, such that the average in-plane stress over the entire superlattice period approaches zero. This approach theoretically allows for the infinite periodic stacking of layers without the risk of strain relaxation. Specifically, for a repeating unit consisting of well and barrier layers, the strain-balance condition can be expressed by the following equation [43]:

(14) $$a_{0}=\frac{A_{W}t_{w}a_{w}a_{b}^{2}+A_{b}t_{b}a_{b}a_{w}^{2}}{A_{W}t_{w}a_{b}^{2}+A_{b}t_{b}a_{w}^{2}}$$

(15) $$A=C_{11}+C_{12}-2\frac{C_{12}^{2}}{C_{11}}$$

where *t_w_* and *t_b_* represent the thicknesses of the well and barrier, respectively, and *A* denotes the biaxial elastic coefficient of the material.

**Figure 13:**
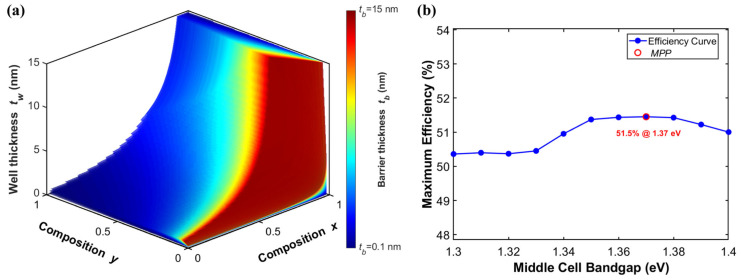
(**a**) Quantum well thickness combinations of Ga*_x_*In_1−*x*_As/GaAs_1−*y*_P*_y_* under composition-dependent strain-balanced conditions; (**b**) Efficiency curve within the tunable bandgap range.

Under the dual constraints of *h_c_* and strain-balancing criteria, this study systematically designs the Ga*_x_*In_1−*x*_As/GaAs_1−*y*_P*_y_* MQWs. Figure 13a presents the combinations of well and barrier thicknesses that satisfy the strain-balancing condition for different material compositions. Excessively thick quantum wells or barriers can significantly hinder carrier tunneling transport. It also increases the probability of carriers being localized within the wells, thereby exacerbating non-radiative recombination losses. These occur even when the strain-balancing condition is satisfied. From the growth process perspective, epitaxial growth of thick layers imposes stringent requirements on the stability of growth rates. Any minor drift in growth parameters can be amplified in thick layers, causing the actual composition to deviate from the design target. Such deviations may disrupt the strain balance and even induce strain relaxation. Therefore, to simultaneously consider material quality, carrier transport properties, and process feasibility, this study restricts the thicknesses of both quantum wells and barriers to within 15 nm. This ensures that bandgap tuning is achieved while maintaining excellent optoelectronic performance and structural stability. As shown in Figure 13b, an optimal middle-cell bandgap of 1.37 eV was identified for the triple-junction solar cell within the tunable range of the quantum well system, corresponding to a conversion efficiency of 51.5%.

In the design of quantum wells, strain engineering and the quantum confinement effect serve as the core physical mechanisms for tuning their optoelectronic properties. For the strain-balanced Ga*_x_*In_1−*x*_As/GaAs_1−*y*_P*_y_* quantum well system grown on a GaAs substrate, the effective bandgap ($E_{g}^{\mathrm{eff}}$) is determined by both the strain-corrected intrinsic bandgap of the quantum well material and the quantum confinement energy. Compressive strain induces splitting of the valence band edge in the quantum well, lowering the energy of the heavy-hole band and thereby reducing its intrinsic bandgap [19]. Furthermore, when carriers are confined within a nanoscale potential well, their motional dimensionality is reduced, resulting in the formation of discrete quantized energy levels (subbands) in the confinement direction [46]. The ground-state energy of the carriers becomes elevated compared to the band edge of the bulk material. This quantum confinement effect significantly increases the effective bandgap of the system. Additionally, sufficiently high potential barriers effectively confine photogenerated carriers within the wells, suppressing their leakage via thermal emission or tunneling. This ensures efficient carrier collection, which is essential for maintaining a high *V_oc_* in the solar cell.

This study selected Ga_0.96_In_0.04_As (8.3 nm) as the quantum well layer and GaAs_0.77_P_0.23_ (3.3 nm) as the barrier layer. This material system offers several key advantages. Its strain-balanced design ensures structural stability and enables high-quality crystal growth. Figure 13b demonstrates that, across the achievable bandgap range of this quantum well system, a bandgap energy of 1.37 eV yields the theoretical maximum efficiency for the triple-junction solar cell. Theoretical calculations indicate that the effective bandgap of this quantum well system can be tuned to approximately 1.37 eV. This bandgap value effectively extends the cell’s absorption range to longer-wavelength photons (~905 nm), thereby enhancing the *J_sc_*. Furthermore, the GaAsP barrier layer provides a sufficient band offset, creating a carrier confinement environment that helps suppress non-radiative recombination within the quantum well. This minimizes the associated *V_oc_* loss. By successfully broadening the spectral response range, this system provides a viable solution for developing triple-junction tandem solar cells with higher *Eff*.

**Figure 14:**
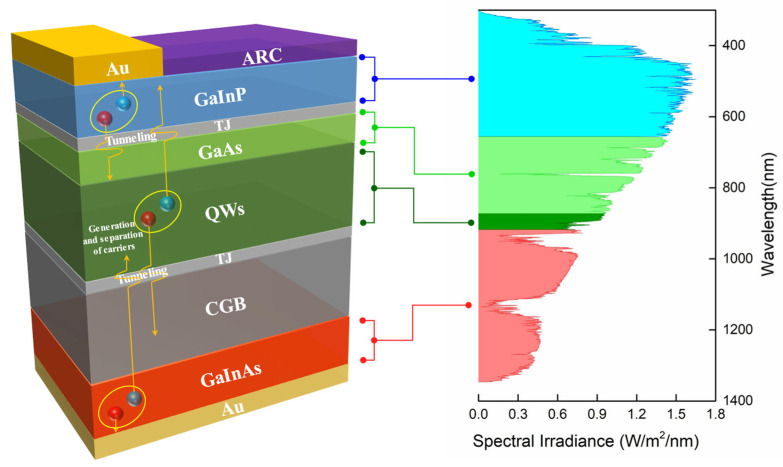
Detailed structure of a triple-junction solar cell and the corresponding spectral absorption characteristics of each subcell.

Based on the design concept described above, Figure 14 illustrates the detailed structure of a tandem III–V triple-junction solar cell. The top, middle, and bottom subcells employ Ga_0.58_In_0.42_P (1.90 eV), GaAs–Ga_0.96_In_0.04_As/GaAs_0.77_P_0.23_ QW (1.37 eV), and Ga_0.63_In_0.37_As (0.93 eV), respectively. This architecture enables graded utilization of the solar spectrum through subcells with different bandgaps. It allows each to absorb photons in its target wavelength range, thereby maximizing overall spectral utilization and achieving a conversion efficiency of 51.5%. To ensure high performance, an anti-reflection coating is applied to reduce surface optical reflection and enhance light coupling. Tunnel junctions are utilized to provide low-loss ohmic interconnections between subcells and facilitate efficient carrier transport. Compositionally graded buffer layers are employed to mitigate the lattice mismatch between the middle and bottom subcells, suppressing the propagation of dislocation defects and preserving crystalline quality. The synergistic effect of these structures forms a critical foundation for realizing high-efficiency multijunction solar cells.

### 3.4. Calculation of the SQ Efficiency Limit for Multijunction Solar Cells

**Figure 15:**
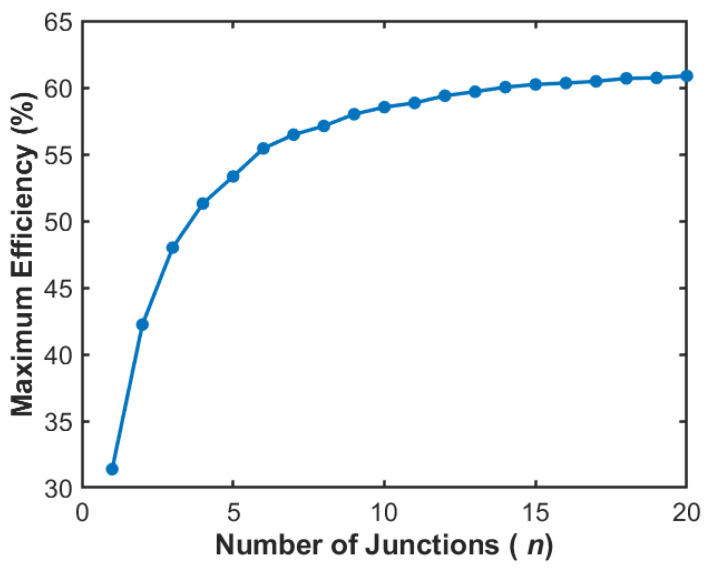
Relationship between the theoretical *Eff_max_* and the number of junctions in tandem solar cells.

A common strategy to further approach the theoretical limit is to increase the number of junctions, enabling a finer division of the solar spectrum and improved photon utilization efficiency. However, as the number of junctions *n* increases, the photon flux absorbed by each subcell is approximately reduced to 1/*n* of that when operating independently. Consequently, for the *n*-th semiconductor in a tandem architecture, its practical maximum output work per absorbed photon, *Wn*, is reduced by a factor of *kT*ln(*n*) [3]. Increasing the number of junctions also leads to diminishing marginal returns in efficiency improvement. Controlling the quality of epitaxial materials and interfaces becomes more challenging [47]. It significantly increases process complexity. Furthermore, device reliability and cost-effectiveness tend to decrease. Figure 15 illustrates the relationship between the maximum efficiency of multijunction solar cells and the number of junctions, considering the reduction in photon absorption flux. For solar cells with up to six junctions, the maximum efficiency (55.4% at *n* = 6) rises rapidly with the increase in junctions. However, the efficiency gains from further increasing the number of junctions diminish quickly, reaching a theoretical maximum of only 60.9% at *n* = 20. Therefore, it is essential to holistically balance the theoretical benefits of spectral matching against the physical limitations and technical challenges posed by the integration of multiple junctions [48].

## 4. Conclusions

Grounded in the detailed balance principle, this study systematically investigated the ultimate efficiency limits and bandgap optimization strategies for single-junction, two-junction, and multijunction systems. Theoretical models were developed and combined with numerical simulations to determine the efficiency limits of various cell architectures under the AM1.5G spectrum. The Al_0.03_Ga_0.97_As/Ge (1.46 eV/0.67 eV) cell configuration is found to achieve a high theoretical efficiency of 43.0% while maintaining satisfactory lattice matching for two-junction cells. The results demonstrate that introducing a strain-balanced quantum well structure (Ga_0.96_In_0.04_As (8.3 nm)/GaAs_0.77_P_0.23_ (3.3 nm)) into a triple-junction cell enables effective tuning of the middle subcell bandgap. The bandgap combination of 1.90 eV/1.37 eV/0.93 eV enables a significantly improved utilization of the solar spectrum, leading to a theoretical conversion efficiency of 51.5%. This work also evaluates the potential and marginal benefits of increasing the number of junctions beyond three. Considering efficiency, cost, and manufacturability, structures with up to six junctions are deemed more practically viable under current technological conditions. These designs strike an optimal balance between high conversion efficiency (with a six-junction theoretical limit of 60.9%), controllable manufacturing cost, and technical feasibility. The study provides important theoretical support and structural design guidance for developing next-generation high-efficiency solar cells, and future work will involve experimental verification through precise epitaxial growth techniques, such as MOCVD, aimed at materializing the predicted bandgap engineering schemes and quantum well structures.

## Figures and Tables

**Table 1:** Best research-cell efficiencies.

Category	Technology Type	Efficiency	Reference
III–V Multijunction Cells	Two-, three-, and four-junction (concentrator)	47.6%	[8]
	Three-junction or more (non-concentrator)	39.5%	[7]
	Two-junction (non-concentrator)	32.9%	[19]
Single-Junction GaAs	Single crystal	27.8%	[20]
	Concentrator	30.8%	[21]
	Thin-film crystal	29.1%	[22]
Crystalline Si Cells	Single crystal (concentrator)	27.6%	[23]
	Single crystal (non-concentrator)	26.1%	[24]
	Thin-film crystal	21.2%	[25]
Thin-Film Technologies	CIGS (concentrator)	23.3%	[26]
	CIGS	23.6%	[27]
	CdTe	23.1%	[28]
Emerging PV	Dye-sensitized cells	13.0%	[29]
	Perovskite cells	27.0%	[20]
	Organic cells	19.2%	[21]
	CZTSSe cells	15.8%	[30]
	Perovskite tandem cells	30.1%	[31]
Hybrid Tandems (2terminal)	Perovskite/Si	34.9%	[20]
	Perovskite/organic	23.4%	[32]
	III–V/Si	36.1%	[33]

## Data Availability

The original contributions presented in this study are included in the article. Further inquiries can be directed to the corresponding author.
